# Hierarchical Afferent Connectivity Drives Population-Wide Bursting Dynamics in a Computational Model of Human-Derived Excitatory Neuronal Networks

**DOI:** 10.1523/JNEUROSCI.0912-25.2026

**Published:** 2026-03-24

**Authors:** Valerio Barabino, Francesca Callegari, Sergio Martinoia, Paolo Massobrio

**Affiliations:** ^1^Neuroengineering Genoa Group, Department of Informatics, Bioengineering, Robotics and Systems Engineering (DIBRIS), University of Genova, Genova 16145, Italy; ^2^IRCCS Ospedale Policlinico San Martino, Genova 16132, Italy; ^3^National Institute for Nuclear Physics (INFN), Genova 16145, Italy

**Keywords:** connectivity, excitatory neurons, human-induced pluripotent stem cells, in silico network model, in vitro recordings, spontaneous network activity

## Abstract

This work presents a computational model of excitatory neuronal networks derived from human-induced pluripotent stem cells, whose activity was recorded with microelectrode arrays. A key feature of in vitro neuronal cultures is the emergence of network bursts (NBs)—population events involving most neurons, characterized by different durations, firing frequencies, and recruitment patterns. Our numerical approach investigates the mechanisms underlying these dynamics, addressing the limitations of experimental systems that make it difficult to isolate specific parameters and processes. The model aims to investigate how local neuronal dynamics and global structural connectivity interact to shape the emergence, propagation, and termination of NBs, highlighting the interdependence between intrinsic and network-level mechanisms. We demonstrate the critical role of noise in triggering NBs. At the same time, nonrandom, structured network topologies are essential for sustaining and shaping the resulting collective spatiotemporal firing patterns. In particular, we showed that the organization of incoming and outgoing degrees significantly modulates population recruitment and burst structure, with a hierarchical organization of afferent connectivity emerging as the dominant determinant of collective dynamics. By integrating in vitro observations into in silico simulations, the present study provides a solid foundation for understanding the principles governing human neuronal network function. Also, it sets the stage for investigating how alterations of network properties may contribute to pathological conditions.

## Significance Statement

We developed a computational model that replicates the spontaneous activity of neuronal networks derived from human-induced pluripotent stem cells. With a build-to-understand approach, this model helps us to understand how human brain cells generate complex activity patterns and highlights the critical role of noise in triggering collective population events. Our results demonstrate how network topology influences neuronal communication and information processing. By integrating experimental observations and theoretical models, this work provides a new tool for exploring the principles of human brain function and how these processes may be disrupted in neurological conditions.

## Introduction

Human-induced pluripotent stem cell (hiPSC)-derived neuronal networks constitute a powerful in vitro platform in neuroscience because they retain the genetic characteristics of the donor, thereby enabling the study of subject-specific neuronal dynamics and disease mechanisms (Frega et al., 2019; Mossink et al., 2021). When grown on microelectrode arrays (MEAs), these networks can be monitored noninvasively with high temporal resolution, providing access to their collective dynamics (Pelkonen et al., 2021; Zanini et al., 2023). A defining feature of such dynamics, consistently observed in both rodent and human cultures, is the occurrence of network bursts (NBs), i.e., highly synchronized events that recruit most neurons in the culture (Wagenaar et al., 2006; Odawara et al., 2016).

Many aspects of network-wide bursting dynamics remain inaccessible to direct experimental observation. Addressing this limitation requires complementary approaches, including computational modeling, which bridges the gap between empirical observations and theoretical understanding (Callegari et al., 2025). This framework provides insight into how intrinsic neuronal properties, structural connectivity, and pathological alterations interact to shape emergent network activity, extending our understanding beyond what can be measured in vitro (Le Masson et al., 2014; Wei et al., 2014; Mok et al., 2022; Doorn et al., 2023).

In rodent preparations, NBs have been extensively characterized and linked to the absence of external inputs (Wagenaar et al., 2005), as well as to the interplay between local and global dynamics (Bosi et al., 2015; Callegari et al., 2023; Barabino et al., 2024). In hiPSC-derived networks, bursts have likewise been consistently observed and are commonly employed as functional readouts to assess maturation or to compare control and disease conditions (Marchetto et al., 2017; Klein Gunnewiek et al., 2020; Linda et al., 2022; Wang et al., 2022). However, in most of these studies NBs are treated primarily as descriptive markers, with limited exploration of the underlying mechanisms. Only more recently mechanistic investigations have begun to emerge: for example, Doorn et al. (2024) analyzed the fragmentation of NBs in patient-derived neurons, providing novel insights into human-specific bursting dynamics. Despite these efforts, the specific mechanisms governing the initiation, temporal evolution, and termination of NBs in hiPSC-derived networks remain largely underexplored.

The goal of this study was to develop a computational model of excitatory hiPSC-derived neuronal networks to reproduce and dissect their spontaneous bursting dynamics. To this end, we adapted the conductance-based Hodgkin–Huxley (HH) model introduced by Doorn et al. (2023), which was originally developed for the same type of hiPSC-derived excitatory neurons and therefore provided a validated starting point for our investigation. In our implementation, neurons were embedded in a 2D spatial network, where cells were positioned according to densities and spatial distributions derived from experimental observations, thereby capturing biologically plausible constraints. On top of this scaffold, we imposed different connectivity rules to generate distinct topological organizations. Specifically, we investigated four representative architectures: random (RND), small-world (SW), scale-free (SF), and their concurrent existence. The latter was designed to explicitly differentiate between incoming and outgoing connectivity, creating asymmetries absent in the other schemes and that could better reflect the organization of biological networks. Within this framework, we systematically explored how both local parameters (e.g., membrane and synaptic properties) and global properties (e.g., topology, indegree/outdegree distributions) contribute to the initiation, recruitment, and decay of NBs. Our analysis highlights network topology as a critical determinant of burst dynamics, with concurrent architectures emerging as the most consistent with experimentally observed activity patterns. This approach enabled us to compare the relative contributions of parameter tuning and topological constraints, suggesting that while local properties regulate overall activity and bursting levels, the temporal profile of NBs is shaped primarily by topology. By integrating computational modeling with experimental observations, this work advances the mechanistic understanding of spontaneous bursting in hiPSC-derived neuronal networks and establishes a versatile platform to investigate how structural alterations may contribute to pathological dynamics, with potential applications in disease modeling and precision medicine.

## Materials and Methods

**Figure 1.**
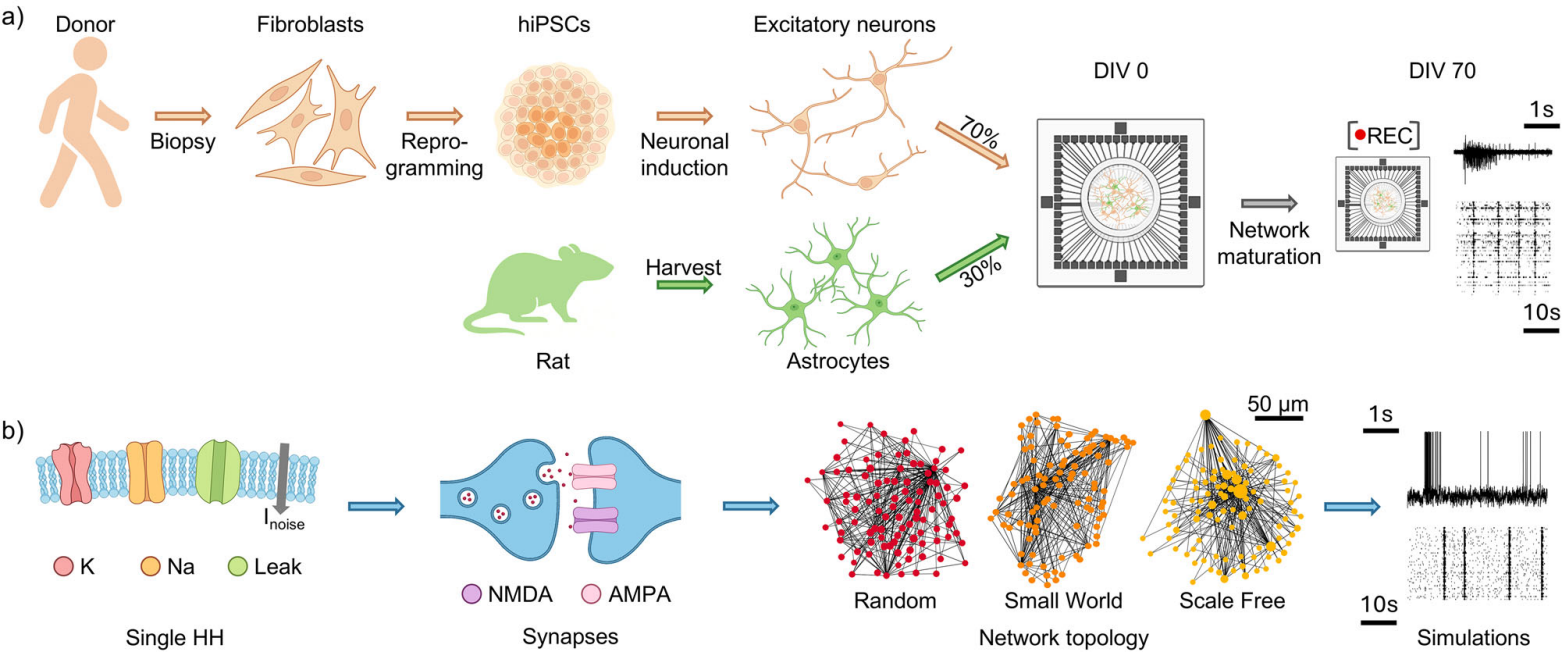
Schematics of the in silico model and the in vitro reference setup. ***a***, Schematics summarizing the experimental protocol used to generate the reference dataset by Parodi et al. (2023). Donor fibroblasts collected from skin biopsies were reprogrammed into hiPSCs. A neuronal induction protocol was then applied to generate excitatory cortical neurons, which were cocultured with rat astrocytes on MEAs to support network maturation. Recordings were performed at a mature stage of development (DIV 70), and spike trains were extracted. This publicly available dataset was used as the experimental reference for analysis and comparison with our in silico model. ***b***, The computational model consists of modified excitatory HH neurons, spatially distributed to reproduce a cell density close to experimental values (∼1,200 cell/mm^2^) and connected with specific topological properties. Parts of the figure were created with Biorender.com.

We devised an in silico model to investigate how network topology and intrinsic neuronal properties shape bursting activity in hiPSC-derived excitatory networks. As experimental reference, we used the publicly available dataset (https://doi.org/10.17605/OSF.IO/SW3BR) from Parodi et al. (2023), which provides long-term MEA recordings of hiPSC-derived neuronal cultures. While Parodi et al. focused on the role of excitation–inhibition balance on the spontaneous development of human-derived neuronal networks in vitro, we restricted our analysis to the 100% excitatory condition at a mature stage of development (∼70 d in vitro). In this configuration, hiPSC were generated from reprogramming donor fibroblasts and hiPSC-derived neurons were obtained by treatment with transcription factors. Neurons were plated on MEAs together with murine astrocytes and recorded under stable culture conditions [Fig. 1*a* for a schematic of the experimental procedure, full details are reported in Parodi et al. (2023)]. The computational model, consisting of 100 modified HH neurons, was adapted from Doorn et al. (2023) to incorporate alternative network topologies (Fig. 1*b*). Parameter values were primarily derived from human experimental data (50%; Table 1); when unavailable, we adopted values from well-validated murine models (42%). The remaining parameters (8%) were fine-tuned using a grid search approach. Parameters classified as “constrained” were explored within ranges derived from experimental or modeling studies, whereas free parameters (i.e., parameters for which no reliable physiological bounds are available in the literature) were explored over broader, unconstrained ranges. In all cases, parameter selection was guided by statistical comparison to experimental metrics to ensure simulations closely reproduced the activity of the biological counterpart. Details on the statistical tests and fitting metrics are provided below, Data analysis and Statistical analysis.

**Table 1.** Neuronal and synaptic parameters used in the presented simulations

Parameter	Description	Value	Origin	Reference
*C_m_*	Specific membrane capacitance	1 µF/cm^2^	Human	(Pospischil et al., 2008; Doorn et al., 2023)
*E_L_*	Nernst potential of leakage	−39.2 mV	Human	(Doorn et al., 2023)
*E_k_*	Nernst potential of potassium ions	−80 mV	Human	(Doorn et al., 2023)
*E*_Na_	Nernst potential of sodium ions	70 mV	Human	(Doorn et al., 2023)
${\bar{g}}_{L}$	Maximum conductance of leakage	0.3 mS/cm^2^	Human	(Pospischil et al., 2008; Doorn et al., 2023)
${\bar{g}}_{K}$	Maximum conductance of potassium channels	5 mS/cm^2^	Human	(Pospischil et al., 2008; Doorn et al., 2023)
${\bar{g}}_{\mathrm{Na}}$	Maximum conductance of sodium channels	50 mS/cm^2^	Human	(Pospischil et al., 2008; Doorn et al., 2023)
*V*_th_	Constant to alter firing threshold	−30.4 mV	Human	(Doorn et al., 2023)
*σ_N_*	Membrane potential oscillation amplitude	5.35 mV	NA	Fitted
*v*_eff_	Effective propagation speed	25 mm/s	Human	(Jacobi and Moses, 2007; Doorn et al., 2023)
*E*_AMPA_	Reversal potential of AMPA channels	0 mV	Murine	(Destexhe et al., 1998)
*E*_NMDA_	Reversal potential of NMDA channels	0 mV	Murine	(Destexhe et al., 1998)
${\bar{g}}_{\mathrm{AMPA}}$	Maximum conductance of AMPA channels	0.35 nS	Murine	Fitted; (Destexhe et al., 1998)
${\bar{g}}_{\mathrm{NMDA}}$	Maximum conductance of NMDA channels	0.0275 nS	Murine	Fitted; (Destexhe et al., 1998)
*τ*_AMPA_	Decay time constant of AMPA channels	2 ms	Human	(Doorn et al., 2023)
*τ*_NMDA_	Decay time constant of NMDA channels	100 ms	Human	(Doorn et al., 2023)
*τ*_NMDA_	Rise time constant of NMDA channels	2 ms	Human	(Doorn et al., 2023)
[Mg^2+^]*_o_*	Extracellular magnesium concentration	1 mM	Murine	(Jahr and Stevens, 1990)
*a*_NMDA_	Constant of NMDA dynamics	0.062 mV^−1^	Murine	(Jahr and Stevens, 1990)
*b*_NMDA_	Constant of NMDA dynamics	3.57 mM	Murine	(Jahr and Stevens, 1990)
*τ*_STD_	Recovery time constant of synaptic vesicles	800 ms	Murine	(Masquelier and Deco, 2013)
*f_D_*	Synaptic depression strength	0.75%	NA	Fitted
${\bar{g}}_{\mathrm{AHP}}$	Maximum conductance of AHP channels	0.0035 nS	Murine	(Masquelier and Deco, 2013)
*τ*_AHP_	Recovery time constant of AHP channels	6 s	Murine	(Masquelier and Deco, 2013)

### Ethicsapproval

No new experiments have been performed for this work. Reference data came from the already published dataset with the doi 10.17605/OSF.IO/SW3BR.

### Neuronal network model

We developed a neuronal network model consisting of 100 neurons, described with the HH formalism as follows (Hodgkin and Huxley, 1952):

$$\begin{array}{rl} & C_{m}\frac{dV_{m}}{dt}\;=\;-{\bar{g}}_{\mathrm{Na}}m^{3}h\;(V_{m}-E_{\mathrm{Na}})-\;{\bar{g}}_{K}\;n^{4}\;(V_{m}-E_{K})-\;{\bar{g}}_{L}(V_{m}-E_{L})\\ & \;-{\bar{g}}_{\mathrm{AHP}}\;s_{\mathrm{AHP}}\;(V_{m}-E_{K})+I_{\mathrm{syn}}+I_{\mathrm{noise}}, \end{array}\;(1)$$

where $V_{m}$ is the membrane potential; $C_{m}$ is the specific membrane capacitance; ${\bar{g}}_{\mathrm{Na}}$, ${\bar{g}}_{K}$, ${\bar{g}}_{L}$, and ${\bar{g}}_{\mathrm{AHP}}$ are the sodium (Na), potassium (K), leakage (L), and afterhyperpolarization (AHP) maximum conductances; $s_{\mathrm{AHP}}$, is the fraction of open AHP channels; $E_{\mathrm{Na}}$, $E_{K}$, and $E_{L}$ are the Nernst potentials; n, m, and h, are the gating variables; $I_{\mathrm{syn}}$ is the synaptic current; and $I_{\mathrm{noise}}$ is a noisy drive. The rate constants of the equations of the model were chosen to align with observations of cortical neurons and are reported in the Supplementary Material (Eqs. S1–S9; Traub et al., 1991). Each neuron consists of a single compartment representing the cell body.

Each neuron features leakage, sodium, and potassium voltage-dependent channels, and an additional AHP channel corresponding to slow calcium- and sodium-activated potassium currents (Eq. S10; Larsson, 2013; Doorn et al., 2023). This potassium current represents neuronal adaptation as an intrinsic fatigue mechanism with a recovery timescale of 6 s (Table 1; Masquelier and Deco, 2013). The Nernst potentials, maximum conductances, and the spike activation threshold were set according to Doorn et al. (2023), who fitted these values to match the shape of simulated action potentials to those of in vitro hiPSC-derived neurons (Table 1).

As in vitro neuronal networks exhibit self-sustained spontaneous activity, we introduced white noise to all cells of the network to generate underthreshold oscillations and random low-frequency spikes in accordance with the following distribution:

$$I_{\mathrm{noise}}\;=\;\sigma_{N}\;\sqrt{2\frac{g_{L}\;C_{m}}{dt}}\;\xi \;,\;(2)$$

where ξ is a normally distributed variable with a mean of 0 and a standard deviation of 1, dt is the simulation time step, and $\sigma_{N}$ is the standard deviation of the noisy fluctuations in the membrane potential (Table 1). Finally, $g_{L}$ and $C_{m}$ are the leakage conductance and the specific membrane capacitance of the neuron, respectively (Table 1).

**Figure 2.**
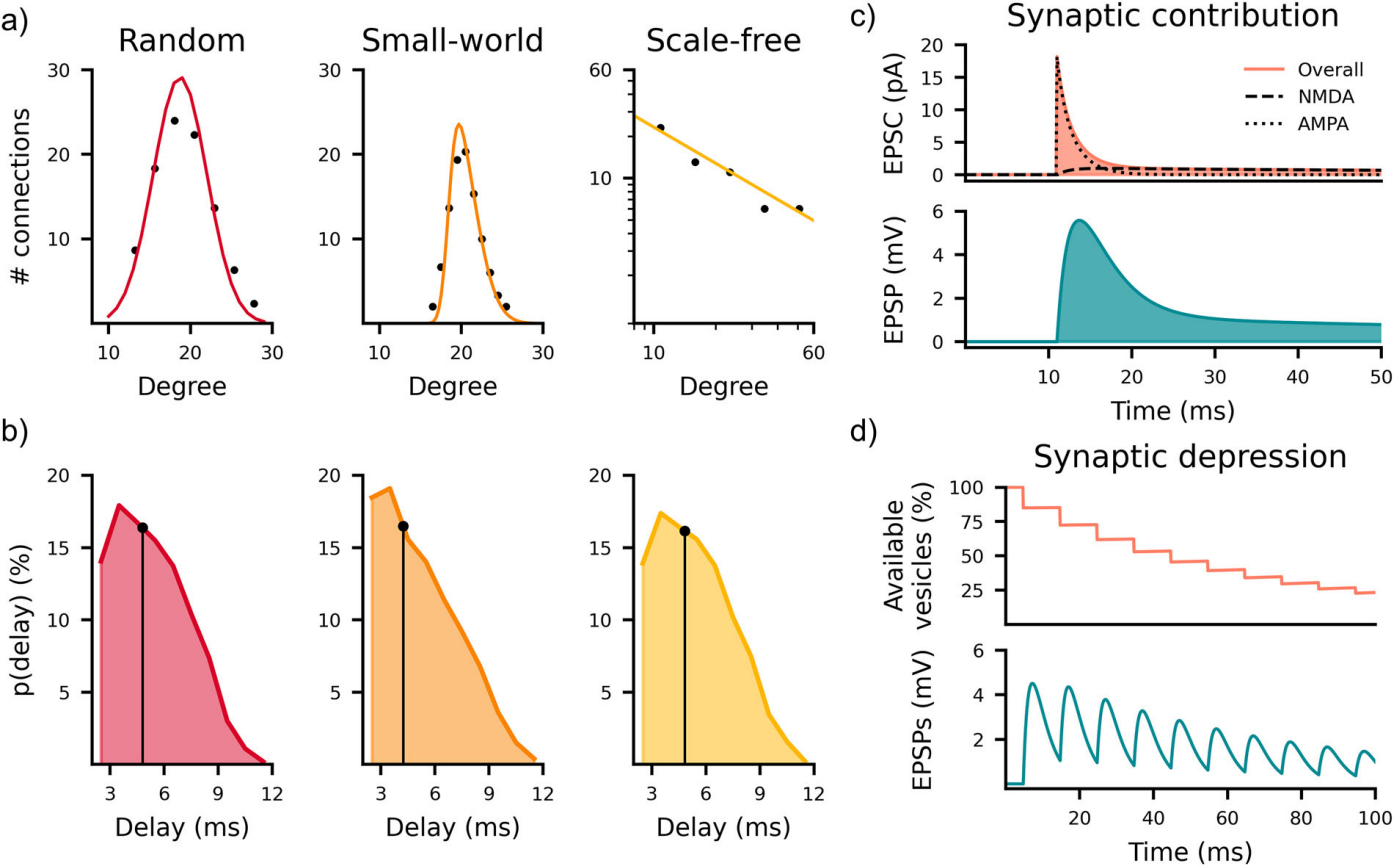
Model dynamical and topological properties. ***a***, Connectivity degree distributions of representative RND, SW, and SF networks. RND networks are characterized by a Gaussian distribution, SW with a skewed distribution (*p_rw_* = 30%), and SF with a power law relationship (*α*_SF_ = 2). ***b***, Synaptic delay distributions due to the spatial arrangement of the network. The black line represents the average value for each topology. ***c***, Postsynaptic current (up) and potential (down), featuring fast AMPA and slow NMDA contributions. ***d***, Short-term synaptic depression modeled according to Abbot’s paradigm: after each presynaptic spike, the number of ready-to-release vesicles decreases proportionally to the available pool.

The cells were arranged within a 160-µm-radius circle to achieve a cell density ∼1,200 cell/mm^2^ (Fig. 1*b*) in accordance with the experimental procedures (Parodi et al., 2023). The connectivity of the neurons was represented as a directed graph, where nodes correspond to neurons and edges represent synaptic connections between presynaptic and postsynaptic neurons. In such a graph, degree distributions describe the connectivity patterns, distinguishing between indegree (incoming connections) and outdegree (outgoing connections). Accordingly, we tested three well-known topologies both for incoming and outgoing degree: RND, SW, and SF. The RND topology is characterized by a Gaussian distribution (Fig. 2*a*, left). SW networks are mechanistically built from a ring topology (lattice), and each connection has a probability of being rewired. In the simulations, we set such a value at $p_{rw}=30\text{\%}$. The resulting degree distribution is a skewed Gaussian distribution (Fig. 2*a*, center). Lastly, SF networks are characterized by a power law distribution in the logarithmic domain of the degree distribution (Fig. 2*a*, right). We labeled a topology featuring the same connectivity rule for both the incoming and outgoing connections as “homogeneous” and “concurrent” otherwise. For all topologies, the average connectivity percentage was maintained at ∼20% (Gritsun et al., 2010; Callegari et al., 2023). We did not consider autapses (i.e., self-connections; Liu et al., 2009). It is important to note that while the connectivity rules are applied topologically, the neurons are embedded in a two-dimensional Euclidean space. We implemented spatially dependent synaptic delays $\left(\Delta_{T}\right)$ based on the distance between neurons, adopting a phenomenological approach in which no additional fixed synaptic delay is introduced, and network processing is implicitly captured by an effective propagation speed. Following the approach of Doorn et al. (2023), transmission delays were computed as the ratio of the interneuronal distance with the activity-front propagation speed (kept constant in all the simulations and set to 25 mm/s; Table 1; Jacobi and Moses, 2007; Doorn et al., 2023), as follows:

$$\Delta_{T}=\frac{d(x,y)}{v_{\mathrm{eff}}},\;(3)$$

where d(x,y) is the Euclidean distance between neurons and $v_{\mathrm{eff}}$ is the effective propagation speed. Depending on the modes of connectivity, we estimated the average transmission delay $\left(\Delta_{T}\right)$ for each configuration (Fig. 2*b*). Both RND and SF networks exhibited an average delay of $\Delta_{T}=4.8\;\mathrm{ms}$, while SW networks featured a slightly shorter delay of $\Delta_{T}=4.2\;\mathrm{ms}$. In any case, simulations display a maximum delay of ∼13 ms. Movie S1 shows the simulated spatiotemporal electrophysiological propagation (“top”), compared with a representative experiment (“bottom”).

**Movie 1.** Spatiotemporal propagation of a simulated neuronal network (***a***) compared with a representative experiment (***b***). [View online]

The excitatory synaptic transmission $\left(I_{\mathrm{syn}}\right)$ has been split into fast AMPA $\left(I_{\mathrm{AMPA}}\right)$ and slow NMDA $\left(I_{\mathrm{NMDA}}\right)$ components, in accordance with the experimental characterizations (Destexhe et al., 1998). Each excitatory component is described following the formalism for chemical transmission (Fig. 2*c*). The following relationships were adopted:

$$I_{\mathrm{syn}}=\;I_{\mathrm{AMPA}}+\;I_{\mathrm{NMDA}},\;(4)$$

$$I_{\mathrm{AMPA}}=\;{\bar{g}}_{\mathrm{AMPA}}\;(E_{\mathrm{AMPA}}-\;V_{m})\;\underset{k}{\sum }\;w_{k}^{\mathrm{syn}}s_{k}^{\mathrm{AMPA}},\;(5)$$

$$I_{\mathrm{NMDA}}=\;{\bar{g}}_{\mathrm{NMDA}}\;u(V_{m})\;(E_{\mathrm{NMDA}}-V_{m})\;\underset{k}{\sum }\;w_{k}^{\mathrm{syn}}s_{k}^{\mathrm{NMDA}},\;(6)$$

where ${\bar{g}}_{\mathrm{AMPA}}$ and ${\bar{g}}_{\mathrm{NMDA}}$ are the maximal conductances, $E_{\mathrm{AMPA}}$ and $E_{\mathrm{NMDA}}$ are the synaptic reversal potentials, $V_{m}$ is the postsynaptic membrane potential, and $s^{\mathrm{AMPA}}$ and $s^{\mathrm{NMDA}}$ are the fraction of open channels for AMPA and NMDA synapses, respectively. The sums over k represent all the spikes emitted by the presynaptic neuron k, and $w_{k}^{\mathrm{syn}}$ is the synaptic weight subject to short-term plasticity. The NMDA conductance (Eq. 6) is voltage-dependent as the fraction of NMDA receptors unblocked (u) by magnesium ions depends on the postsynaptic potential and is fitted as follows:

$$u(V_{m})=\frac{1}{1+e^{-a_{\mathrm{NMDA}}V_{m}}\;({\left[Mg^{2+}\right]}_{o}/b_{\mathrm{NMDA}})},\;(7)$$

where $a_{\mathrm{NMDA}}$ and $b_{\mathrm{NMDA}}$ are fitting parameters, set to 0.062 mV^−1^ and 3.57 mM, respectively (Jahr and Stevens, 1990). We assumed that the magnesium block changes instantaneously and depends on the extracellular magnesium concentration $[Mg^{2+}{]}_{o}$ set to 1 mM. The fractions of open channels $s^{\mathrm{AMPA}}$ and $s^{\mathrm{NMDA}}$ are as follows:

$$\frac{ds_{k}^{\mathrm{AMPA}}}{dt}=\;-\frac{s_{k}^{\mathrm{AMPA}}}{\tau_{\mathrm{AMPA}}}\;+\underset{s}{\sum }\;\delta (t-t_{s}^{k}-\Delta_{T}),\;(8)$$

$$\frac{ds_{k}^{\mathrm{NMDA}}}{dt}=\;-\frac{s_{k}^{\mathrm{NMDA}}}{\tau_{\mathrm{decay}}^{\mathrm{NMDA}}}+\;\frac{x_{k}^{\mathrm{NMDA}}}{\tau_{\mathrm{rise}}^{\mathrm{NMDA}}}\;(1-s_{k}^{\mathrm{NMDA}}),\;(9)$$

$$\frac{dx_{k}^{\mathrm{NMDA}}}{dt}=\;-\frac{x_{k}^{\mathrm{NMDA}}}{\tau_{\mathrm{rise}}^{\mathrm{NMDA}}}+\underset{s}{\sum }\;\delta (t-t_{s}^{k}-\Delta_{T}).\;(10)$$

AMPA rise was considered practically instantaneous (i.e., <<1 ms; Masquelier and Deco, 2013), while NMDA rise was set at 2 ms $\left(\tau_{\mathrm{rise}}^{\mathrm{NMDA}}\right)$. The decay time constants $\tau_{\mathrm{decay}}^{\mathrm{AMPA}}$ and $\tau_{\mathrm{decay}}^{\mathrm{NMDA}}$ were set at 2 and 100 ms, respectively. $x_{k}^{\mathrm{NMDA}}$ is an auxiliary gating variable to account for the dynamics of opening of NMDA receptors. The sum over s represents the spikes emitted by the presynaptic neuron k at times s. $\Delta_{T}$ is the distant-dependent delay. We tested different values for both AMPA and NMDA maximum conductance ( ${\bar{g}}_{\mathrm{AMPA}}$, ${\bar{g}}_{\mathrm{NMDA}}$) according to the ranges reported in Destexhe et al. (1998; Table 1). Their values will be further discussed in Results, Balance between synaptic conductance and depletion is responsible for an in vitro-like bursting activity.

Each synapse features short-term depression (STD), modeled according to Abbott’s paradigm (Dayan and Abbott, 2005). In each presynaptic terminal, there is a pool of ready-to-release vesicles. After each release, the number of available vesicles decreases (Fig. 2*d*). The generation of new ready-to-release vesicles has a time course from hundreds of milliseconds up to seconds. After the arrival of each spike at the presynaptic terminal, the number of ready-to-release vesicles decreases by a factor of $f_{D}$, representing the magnitude of depression. This plasticity process is described by a first-order differential equation, which is integrated at each simulation step to update synaptic efficacy dynamically:

$$\frac{dw_{k}^{\mathrm{syn}}}{dt}=\;\frac{1-w_{k}^{\mathrm{syn}}}{\tau_{\mathrm{STD}}}\;-f_{D}\;w_{k}^{\mathrm{syn}}\underset{s}{\sum }\;\delta (t-t_{s}^{k}-\Delta_{T}),\;(11)$$

where $w_{k}^{\mathrm{syn}}$ represents the fraction of available synaptic resources for presynaptic neuron k, dynamically modulated by the STD mechanism. After a decrease in the number of vesicles, $w_{k}^{\mathrm{syn}}$ returns to 1 with a time constant of $\tau_{\mathrm{STD}}=800\;\mathrm{ms}$.

### Simulation protocol

All the networks were simulated for 305 s (the first 5 s were discarded as transient period), unless otherwise specified. The models were developed with Brian2 (2.5.1) simulator (Stimberg et al., 2019), in the Python language (3.10.11; van Rossum, 1995). Differential equations were integrated numerically with Euler method for neuron dynamics and exponential Euler method for synapses, with a time step dt of 100 µs. The choice of a 100 µs timestep represents a valid compromise between computational efficacy and simulation fidelity, ensuring accurate waveform shape (Fig. S1*a*), timing accuracy (Fig. S1*b*), and long-term numerical stability (Fig. S1*c*–*e*).

### Data analysis

Both simulated and experimental data were analyzed with in-house Python code inspired by SpyCode (Bologna et al., 2010). For comparison with experimental MEA recordings, we computed population-level measures of network activity, including mean firing rates (MFR), burst durations (BD), and cumulative instantaneous firing rates (IFR), over the entire simulated network. This approach captures the collective dynamics observed experimentally and avoids the need for a direct neuron-to-electrode mapping or virtual subsampling, which would not alter the statistical properties of the measured signals.

#### Spiking, bursting, and network bursting analysis

A simulated spike was detected when the membrane potential reached 0 mV. From the spike train generated by the simulator, we computed the MFR, i.e., the mean number of spikes per second (spikes/s). A neuron was considered active if its MFR was >0.1 spikes/s. From the spike trains, bursts were detected using the method presented in Pasquale et al. (2010). A burst was detected if it was composed of at least 10 spikes with a maximum intraburst interspike interval of 50 ms. Bursts were discarded if they had a mean frequency intraburst lower than 50 spikes/s. The mean bursting rate (MBR) was calculated averaging the mean number of bursts per minute (bursts/min) of each bursting neuron/electrode. A neuron was considered bursting if its MBR was >0.4 bursts/min (Mossink et al., 2021). For each neuron, we extracted the following metrics, averaged over all the detected burst events: the BD, i.e., the duration of the burst events and the number of spikes per burst (SpB). We directly compared each metric with the in vitro experimental counterpart.

To identify network bursting activity, we exploited the algorithm developed by Van Pelt et al. (2004). We computed the cumulative IFR, by convolving the cumulative spike train with a rectangular step function, with a time window of 100 ms. To detect the onset and offset of NBs, the cumulative IFR was multiplied by the number of active neurons/electrodes in 25 ms bins. In this way, we ensure that the activity periods display a sufficiently high spiking frequency as well as a broad electrode involvement (i.e., spatial recruitment). An NB was detected if the product exceeds a threshold set to 5% of the maximum obtained value (Van Pelt et al., 2004). To guarantee the separation among NBs, the minimum inter-NB interval was set to 800 ms.

From the NB events, we computed the spike time histogram (STH) using the cumulative IFR (before the multiplication by the number of active electrodes), averaged over all aligned bursts to capture the temporal profile of network activity. We isolated the cumulative IFR of each NB i, from 500 ms before the event beginning $\left(t_{i}^{\mathrm{start}}\right)$ up to the maximum detected network BD $(\mathrm{NB}D_{\max})$ to guarantee that all NB profiles had the same length. We temporally aligned all NB profiles according to a shift given by calculating the correlation of each event with a reference event, chosen as the one with the highest mean correlation value. The STH was computed as the average profile of all aligned traces. The temporal evolution of the STH was analyzed focusing on the slope of the rising and decaying phases of the obtained profiles (fitting the curve between 10 and 80% of the peak amplitude). The rising phase was fitted with a double exponential function as follows (Van De Vijver et al., 2019):

$$\mathrm{ST}H_{\mathrm{rise}}(t)=a_{1}^{\mathrm{STH}}e^{\frac{t}{\tau_{1}^{\mathrm{STH}}}}+a_{2}^{\mathrm{STH}}e^{\frac{t}{\tau_{2}^{\mathrm{STH}}}}+b_{\mathrm{STH}},\;(12a)$$

where the smaller between $\tau_{1}^{\mathrm{STH}}$and $\tau_{2}^{\mathrm{STH}}$ represents the neuron recruitment rate $\left(\tau_{\mathrm{rise}}^{\mathrm{STH}}\right)$. The decay phase was fitted with a single exponential decay as follows (Marom and Shahaf, 2002; Eytan and Marom, 2006):

$$\mathrm{ST}H_{\mathrm{decay}}(t)=c_{\mathrm{STH}}e^{-\;\frac{t}{\tau_{\mathrm{decay}}^{\mathrm{STH}}}}+d_{\mathrm{STH}},\;(12b)$$

where $\tau_{\mathrm{decay}}^{\mathrm{STH}}$ represents the network modulation during NB events. We evaluated the accuracy of the fitting model by computing the coefficient of determination (*R*^2^). Since NBs are sparse events, simulation time was set to 905 s (of which the first 5 s were discarded as transient) to ensure more robust STH profiles.

#### Principal component analysis

Applying the principal component analysis (PCA) transformation to all IFRs, we extracted the weight of each neuron $\left(w_{n}^{\mathrm{PCA}}\right)$, i.e., the coefficient along the first principal components (PCs) identified by the PCA. This parameter $\left(w_{n}^{\mathrm{PCA}}\right)$ reflected the contribution of each neuron to the overall activity of the network, as the first PC explained most of the variance (Results). We compared $w_{n}^{\mathrm{PCA}}$ to the degree distribution, which showed a saturating, nonlinear trend rather than a linear relationship (Results). To capture this behavior, we fitted $w_{n}^{\mathrm{PCA}}$ using a sigmoid function as follows:

$$w_{n}^{\mathrm{PCA}}(\deg_{n}^{\mathrm{IN}/\mathrm{OUT}})=\frac{a_{\mathrm{sig}}}{b_{\mathrm{sig}}+\;\exp(-c_{\mathrm{sig}}\;*\;\deg_{n}^{\mathrm{IN}/\mathrm{OUT}})},\;(13)$$

where $a_{\mathrm{sig}},\;\;b_{\mathrm{sig}},\;\mathrm{and}\;c_{\mathrm{sig}}$ are fitting parameters and $\deg_{n}^{\mathrm{IN}/\mathrm{OUT}}$ represents the number of incoming/outgoing synaptic connections of neuron n in the network.

### Statistical analysis

We evaluated the normal distribution of the data using the Shapiro–Wilk normality test. Since normality was often not ensured, we performed a nonparametric Mann–Whitney *U* test. For analyses involving multiple comparisons within the same table, all *p* values were treated as belonging to a single family of hypotheses and adjusted using the Benjamini–Hochberg false discovery rate procedure. Statistical significance was determined at *p* < 0.05, using adjusted *p* values where multiple-comparison correction was applied.

### Dataset

We simulated *n* = 6 instances of each network configuration (i.e., topology or parameter set) to evaluate statistical differences with the experimental data. We introduced variability in the simulations by varying the seed. This choice of *n* = 6 networks was motivated by the experimental dataset, which includes *n* = 6 in vitro recordings of excitatory hiPSC cultures at DIV 70.

The in vitro data used as reference are publicly available at the following DOI: 10.17605/OSF.IO/SW3BR.

### Code accessibility

The network model files (Python) and the customized functions (Python) used to analyze both the simulated and experimental (reference) data have been deposited in Zenodo. The DOI of the code reported in this article is 10.5281/zenodo.15371493.

## Results

**Figure 3.**
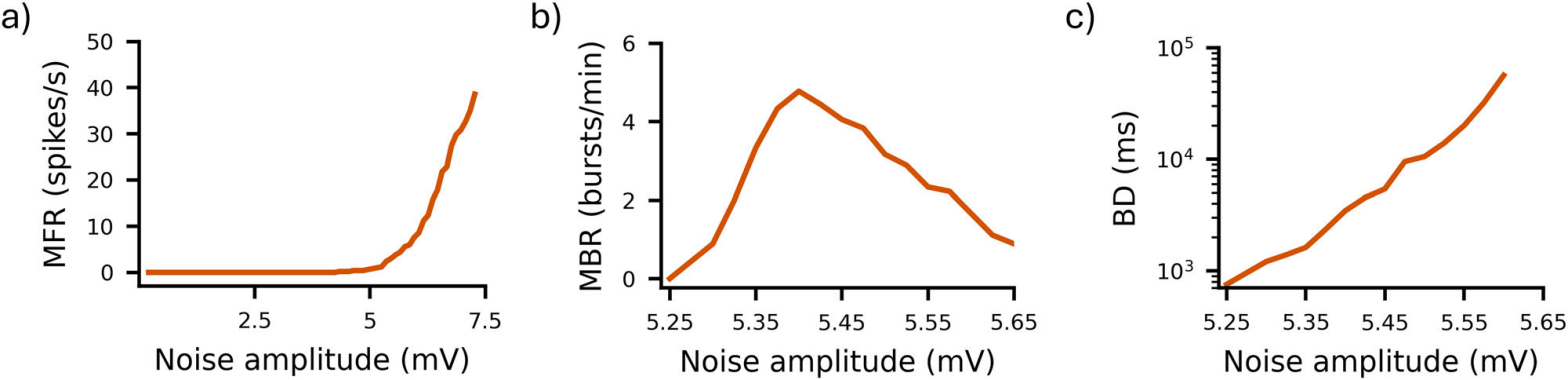
Spiking and bursting activity of the in silico noise-driven networks. ***a***, Gain function as F–V curve (MFR), (***b***) MBR, and (***c***) BD as a function of noise amplitude.

We first assessed how the driving input shapes spontaneous network activity. Different strategies were explored (Figs. S2, S3; Fig. 3). We tested the introduction of a DC current ( $I_{\mathrm{DC}}$ in place of $I_{\mathrm{noise}}$ in Eq. 1; Fig. S2*a*–*d*), and we compared its effect to the noisy current (Eq. 2). DC currents promoted low-frequency spiking activity and synchronized network bursting only within narrow nondifferentiable working region, and only when two different dissipation mechanisms (STD and adaptation) were included (Fig. S2*c*). This high sensitivity to the external drive poses a significant challenge for reproducing in vitro-like activity, which typically shows spontaneous MFR values of ∼1–10 spikes/s (Parodi et al., 2023). Moreover, DC currents could not constrain BD within the target experimental range. Additional tests using pacemaker neurons (Gritsun et al., 2010) yielded qualitatively similar results, indicating that the conclusions on network activity remain largely unchanged compared with the DC current approach (Fig. S2).

The electrophysiological features of hiPSC-derived networks were better captured by noise-driven neurons without adaptation. In murine cultures, noise-driven networks can replicate the key characteristics of in vitro activity without requiring additional features offering a streamlined and flexible modeling approach (Orlandi et al., 2013; Hernández-Navarro et al., 2021). In our simulations, this approach was effective in recapitulating the electrophysiological phenotype observed in human cultures as well. Firing rates increased gradually for noise amplitudes $\sigma_{N}$ in the range [5,6] mV (Fig. 3*a*). Regarding bursting activity, STD was able to extinguish burst events, thus allowing for periods of asynchronous firing. Increasing $\sigma_{N}$ resulted in higher MBR (Fig. 3*b*) up to a critical peak (at $\sigma_{N}\approx 5.4\;\mathrm{mV}$), after which MBR converged to 1 burst/min. Concurrently, we obtained a power law increase in BD (Fig. 3*c*), which could explain the bursting rate bell-shaped curve: stronger inputs yielded fewer but longer bursts. At $\sigma_{N}\approx 5.35\;\mathrm{mV}$, single-channel bursting metrics (MBR ≈ 3.5 bursts/min; BD ≈ 1,180 ms) closely matched experimental data [MBR = 3.5 ± 1.2 bursts/min; BD = 1,090 ± 263 ms; Parodi et al. (2023)]. At the network level, noise-driven networks without adaptation produced higher randomness and greater variability, better aligning with the activity observed in vitro. Therefore, to model low frequencies (MFR ∼10 spikes/s) and less stereotyped activity, all the networks hereafter are noise-driven (with $\sigma_{N}\;=5.35\;\mathrm{mV}$) and without neuronal adaptation.

### Balance between synaptic conductance and depletion is responsible for an in vitro-like bursting activity

Previously, we analyzed how the network’s drive influenced qualitative features, such as the presence of bursting behavior or the variability of inter-NB intervals. In this section, we refined the final subset of free parameters (synaptic conductance and depression magnitude) to accurately reproduce all the single-channel considered metrics (i.e., MFR, MBR, BD, and SpB). Generally, we found that excitatory synaptic transmission played a key role in fueling and sustaining bursting activity, while dissipative mechanisms were responsible for its quenching. Therefore, it was essential to balance and compare these two opposing influences.

Excitatory synaptic currents consist of two primary components: a rapid and usually stronger component mediated by AMPA receptors and a slower, steadier component associated with NMDA receptors (Destexhe et al., 1998). However, for hiPSC-derived neurons, the maximum conductance of these receptors has yet to be experimentally characterized (Roth and van Rossum, 2009; Doorn et al., 2023). In our implementation, we evaluated the ranges of values estimated for murine neurons (Destexhe et al., 1998) to determine their applicability to human cells.

As the primary mediator of excitatory synaptic transmission, changes in AMPA conductance can significantly alter the dynamics of network activity. An increase in AMPA conductance $\left({\bar{g}}_{\mathrm{AMPA}}\right)$ had drastic effects on both the firing and bursting rates (Fig. S4). Even a slight increase from the minimum value of the identified physiological range (0.35 nS; Table 1) resulted in non-in vitro-like high–frequency firing. To preserve realistic, in vitro-like dynamics and to prevent the emergence of unstable regimes during the parameter space exploration, this value was fixed to the lower bound of the physiological range found in Destexhe et al. (1998; Fig. S4). This choice implies that ${\bar{g}}_{\mathrm{AMPA}}$ was not considered for further testing and balance evaluation. On the other hand, the networks had a lower sensitivity to NMDA maximum conductance, allowing for greater operability. An increase in the conductance of this slower and steadier synaptic contribution caused an increase in the excitability of the neurons, fueling recurrent excitatory feedback and promoting longer bursts.

Instead, the end of the bursts was governed with the magnitude $\left(f_{D}\right)$ of short-term synaptic depression (STD), which accounts for the depletion of synaptic vesicles. The range to test the effect of $f_{D}$ was set around the value identified for human cells in Doorn et al. (2023). It causes the progressive reduction in EPSPs amplitude (Fig. 2*d*), leading to a slow decrease in the IFR and, thus, quenching the bursting activity and favoring shorter bursts.

**Figure 4.**
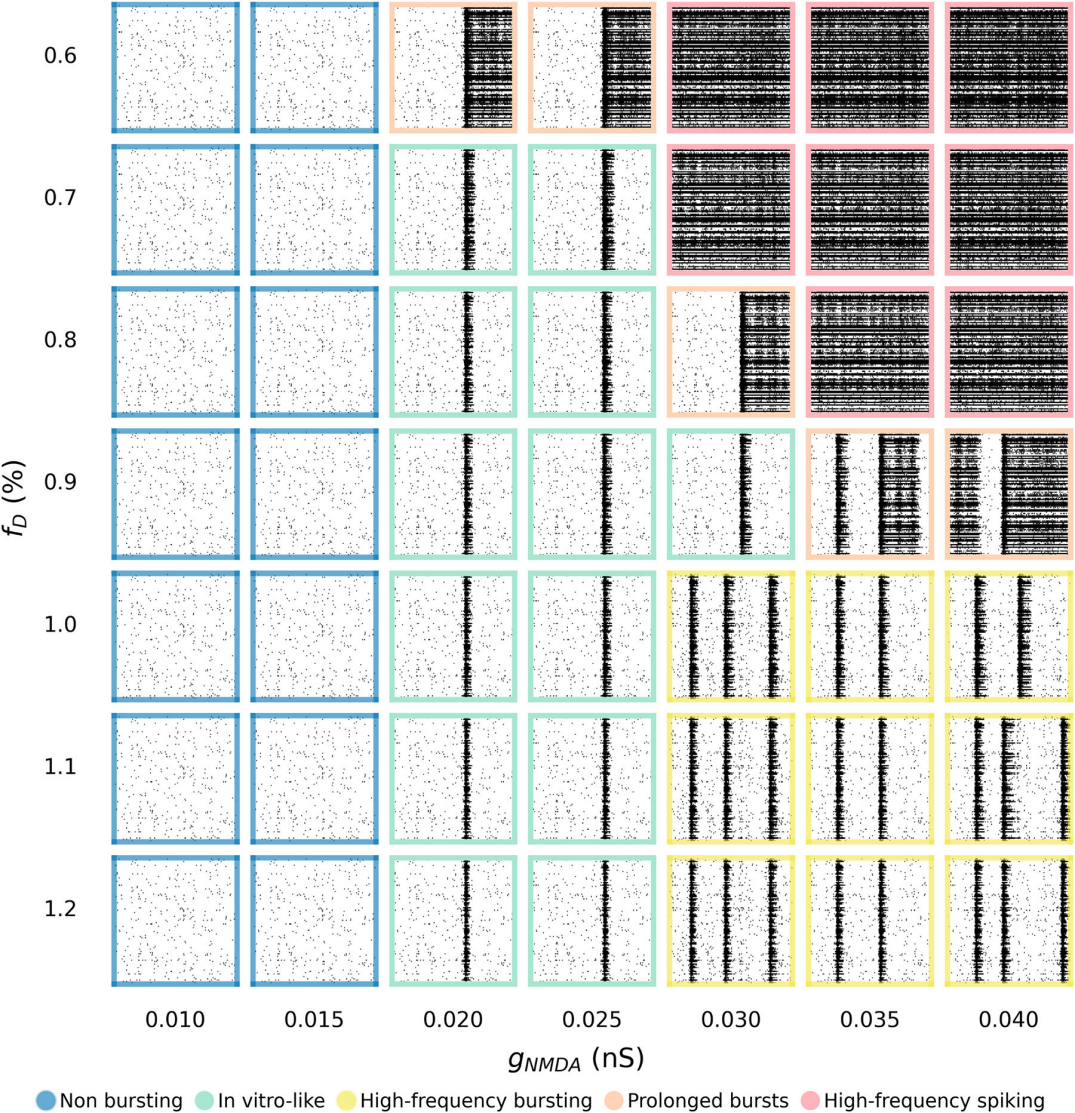
Raster plots of networks with different combinations of synaptic depression magnitude (*f_D_*) and maximum NMDA synaptic conductance (g_NMDA_). Qualitative representation of the states of the network as a function of different values of these two parameters. The time window of each raster plot is 10 s.

We investigated the complementary roles and interactions of these two features, ${\bar{g}}_{\mathrm{NMDA}}$ and $f_{D}$, to achieve a better fit compared with the in vitro observations. By varying their values within the specified ranges (i.e., 0.6% ≤ $f_{D}$ ≤ 1.2% and $0.01\;\mathrm{nS}\leq {\bar{g}}_{\mathrm{NMDA}}\leq 0.04\;\mathrm{nS}$), we identified different network dynamical regimes using a grid search approach (Fig. 4). For low levels of synaptic conductance $({\bar{g}}_{\mathrm{NMDA}}<0.02\;\mathrm{nS})$, the excitatory feedback was insufficient to generate organized activity, resulting in a nonbursting regime characterized by low-frequency asynchronous spiking (Fig. 4, blue region), the typical signature of immature and low connected in vitro networks (Wagenaar et al., 2006). Increasing the synaptic conductance (for $0.02\;\mathrm{nS}\leq {\bar{g}}_{\mathrm{NMDA}}<0.03\;\mathrm{nS}$) resulted in the network displaying a MBR closer to experimental data, ∼4 bursts/min. Under this condition, depression magnitude was able to modulate both the duration and the density of the bursts for *f_D_* ≥ 0.7%. This resulted in an in vitro-like regime of activity, with balanced random spiking and bursting activity (Fig. 4, green region). For ${\bar{g}}_{\mathrm{NMDA}}\geq 0.03\;\mathrm{nS}$, excitability increased, leading to high-frequency spiking. In this case, if the synaptic depression was strong enough (*f_D_* ≥ 1%), the two contributions balanced to display both random spiking and high-frequency bursts with MBR >10 bursts/min (Fig. 4, yellow region). With lower values of depression (*f_D_* ≤ 0.8%), the extinguishing force was not strong enough to silence the burst for long periods (Fig. 4, orange region) or to even allow for the alternation of random spiking and organized burst (Fig. 4, red region). The prolonged-burst dynamical regime (Fig. 4, orange region) also appeared for lower synaptic conductance $(0.02\;\mathrm{nS}\leq {\bar{g}}_{\mathrm{NMDA}}<0.03\;\mathrm{nS})$ and very low synaptic depression (*f_D_* = 0.6%) or for very high synaptic conductance $({\bar{g}}_{\mathrm{NMDA}}>0.03\;\mathrm{nS})$ and intermediate synaptic depression (*f_D_* = 0.9%).

**Figure 5.**
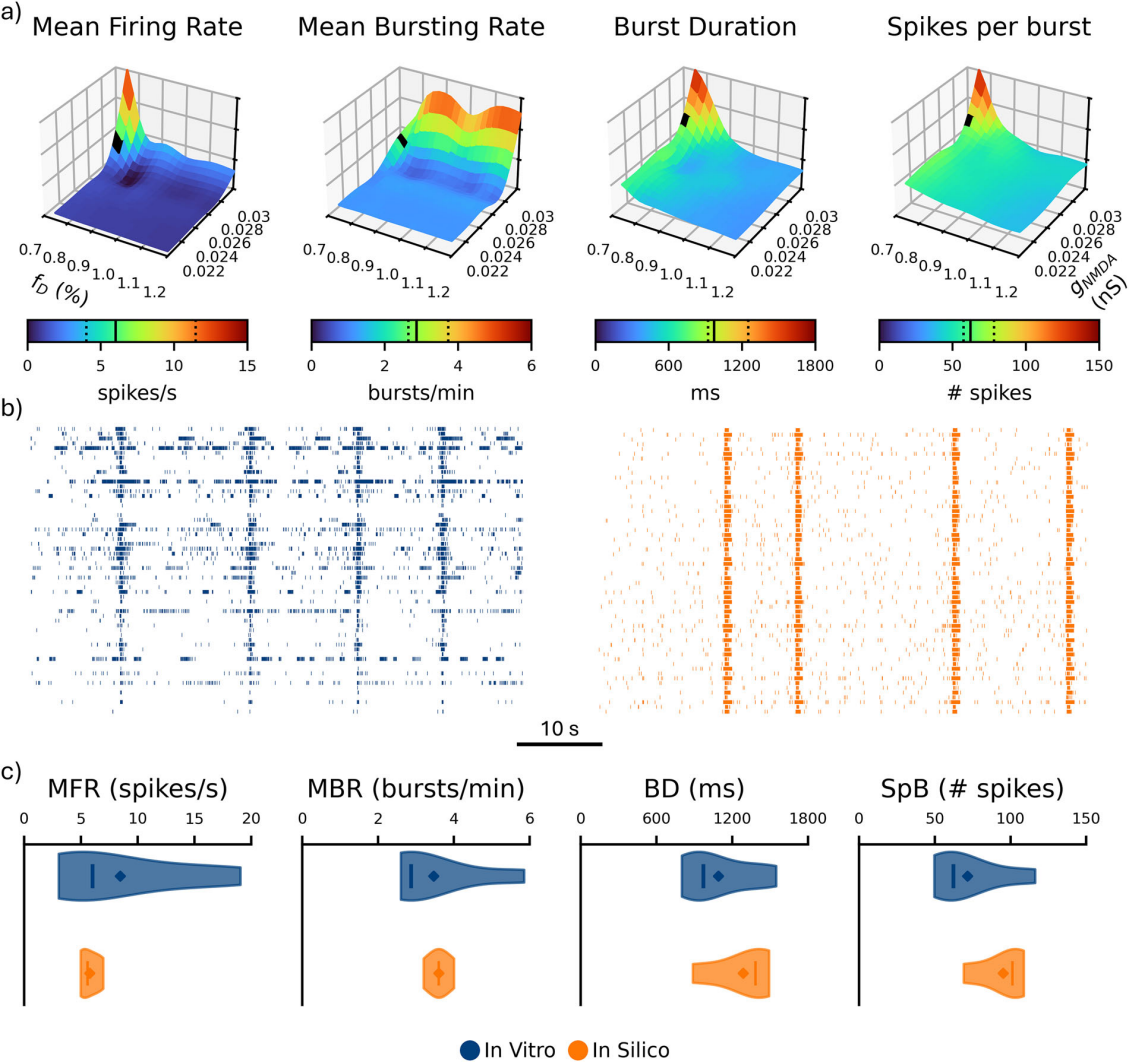
Effect of *g*_NMDA_ and *f_D_* on spiking and bursting activity and comparison with experimental data. ***a***, MFR, MBR, BD, and SpB of the in silico networks as a function of synaptic depression magnitude (*f_D_*) and maximum NMDA synaptic conductance (*g*_NMDA_). The black square indicates the working point selected as the best match to experimental data. The solid and dashed lines in the color bars represent the median and the 25th/75th percentiles of the in vitro data distribution, respectively. ***b***, Raster plots of 60 s of in vitro (blue) and in silico (orange) activity with fixed *f_D_* = 0.75% and *g*_NMDA_ = 0.0275 nS (defined working point). ***c***, Violin plots of the same four metrics shown panel (***a***) of in vitro (blue) and in silico (orange) networks at the chosen working point. The solid line and the diamond represent the median and the mean, respectively. A Mann–Whitney test was performed between the two groups for each metric.

Generally, from these observations, we identified a coarse range of values that corresponded to a dynamical regime consistent with in vitro observations (Fig. 4, green region). To determine the values that better reproduce the firing and bursting metrics of the cultures, we finely sampled this region and its borders (i.e., 0.7% < *f_D_* < 1.2% and $0.02\;\mathrm{nS}<{\bar{g}}_{\mathrm{NMDA}}<0.03\;\mathrm{nS}$; Fig. 5*a*). Within this range, all metrics increased with ${\bar{g}}_{\mathrm{NMDA}}$ and decreased with $f_{D}$, except for the MBR, which was not particularly affected by $f_{D}$. As previously observed (Fig. 4), deviations in either parameter can alter the network's dynamical regime, shifting from asynchronous spiking to prolonged bursts or in vitro-like activity. The excitatory feedback from NMDA conductance sustained bursts, while STD acted as an effective extinguishing force, ensuring physiological BD and preventing excessive firing.

We identified a working point within the parameter space that generates an activity regime closely resembling the spiking and bursting dynamics observed in vitro. When ${\bar{g}}_{\mathrm{NMDA}}$ = 0.0275 nS and *f_D_* = 0.75% (Fig. 5*a*, black square), our model accurately reproduced the hallmarks of mature neuronal cultures, including a balanced mixture of random spiking (22%) and organized bursting activity, as shown in the raster plots (Fig. 5*b*). Specifically, with this optimal parameter set, our in silico model (Fig. 5*c*, orange) showed no statistically significant difference in the main spiking and bursting metrics compared with the considered experiments (Fig. 5*c*, blue). This indicates that the model effectively captured the essential biophysical mechanisms underlying the observed dynamics in hiPSC-derived neurons. The proximity of this optimal point to the transition region among three different dynamical states (Fig. 4) suggests that slight perturbations, such as synaptic modifications, pharmacological interventions, or developmental changes, can push the network into distinct dynamical regimes.

### Topological organization influences the temporal profile of population-wide burst dynamics

**Figure 6.**
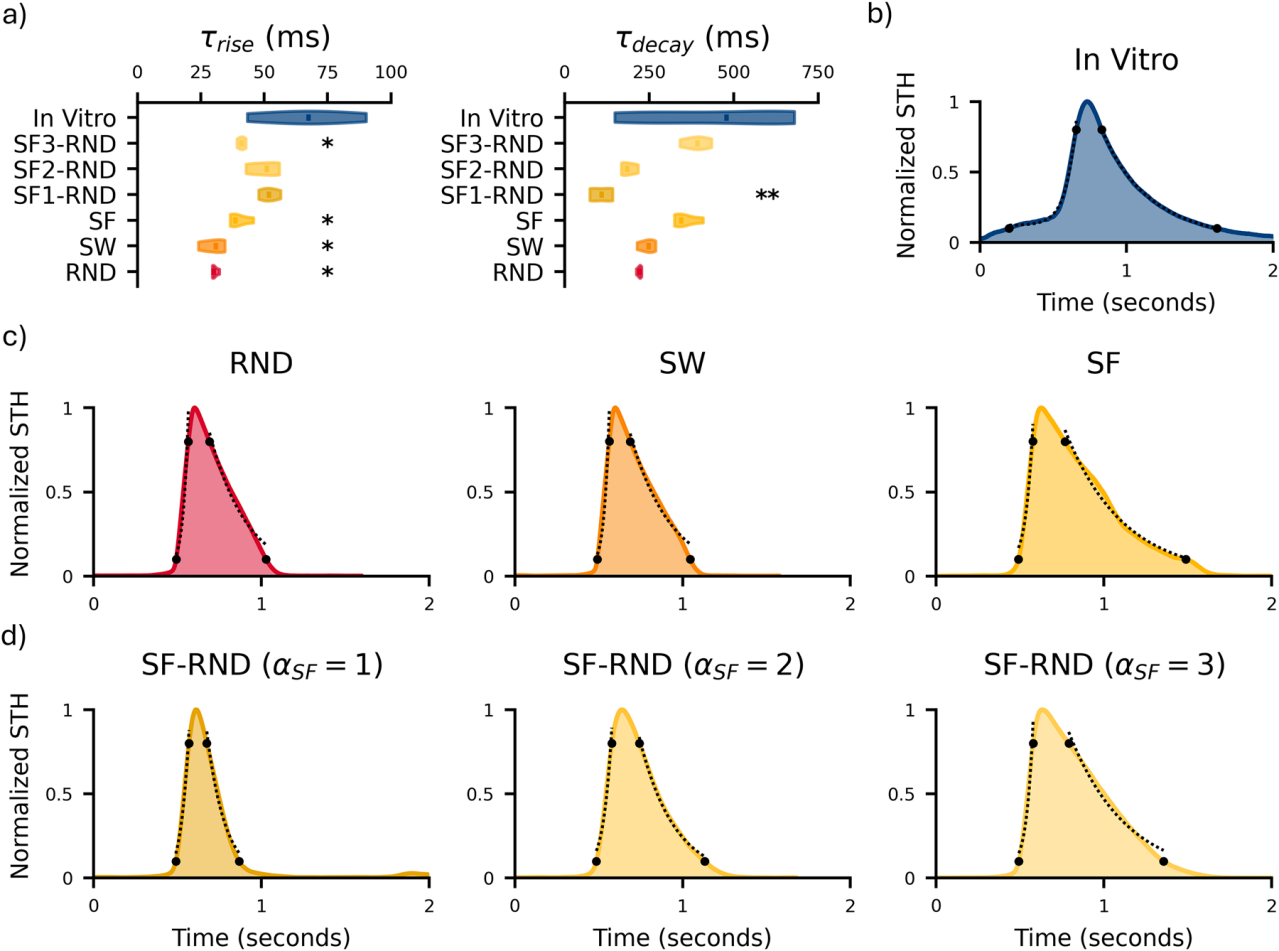
Network activity characterization of in silico networks with different topologies compared with in vitro data. ***a***, Violin plots of the slope of both rise (left) and decay (right) phases of the STH. Mann–Whitney test was performed between each in silico configuration and in vitro (blue) data. * refers to 0.01 < *p* < 0.05 and ** to *p* < 0.01. Normalized STH profiles of (***b***) in vitro data and in silico networks with (***c***) homogeneous topologies, namely, RND, SW, and SF, and (***d***) heterogeneous topologies, featuring incoming SF degree distributions with different slopes (*α*_SF_ = 1, 2, 3) and outgoing RND degree distribution. The dots indicate the limits of the fitting regions for the rise and decay phases. The dotted lines represent the corresponding fits for the rise and decay phases.

In the previous sections, we examined how networks respond to variations in parameters governing local dynamics, such as noise, synaptic conductance, and synaptic depression, to obtain metrics matching those observed in vitro at the single-channel level. However, a critical aspect of such networks is the spatiotemporal integration of neuronal signals, which gives rise to network-wide phenomena characterized by distinctive firing patterns. Their profile exhibits two main phases: a sharp rise of the firing activity, due to the recruitment of the network, followed by a slower relaxation of the network’s activity, whose decay is caused by restoring forces, such as synaptic depression, neuronal adaptation, or the activation of the inhibitory subpopulation (Masquelier and Deco, 2013). Interestingly, the shape of global events, such as NBs, appears to be minimally influenced by local parameters (Brunel and Hakim, 1999; Gritsun et al., 2010). Therefore, to investigate how to modify the profiles of these network events, we introduced specific network topological rules, each imparting unique structural properties with varying organization of the connections. We evaluated three distinct network topologies: RND, SW, and SF. Since our in silico networks were constructed as directed graphs, we are able to distinguish between incoming (afferent) and outgoing (efferent) connections. This distinction allowed us to explore the directional nature of information flow within the networks, providing a more detailed understanding of how the topology influences network dynamics. Given the intrinsic variability of hiPSC-derived networks (Fig. S5), primarily attributable to batch-to-batch differences in both neuronal and astrocytic components (Doorn et al., 2025), we focused our analysis on the shape of STH profiles, normalizing them by their peak firing frequency. This normalization allowed us to account for amplitude differences and focus solely on the temporal dynamics of population activity. This intrinsic variety was reflected in the great variability for both $\tau_{\mathrm{rise}}^{\mathrm{STH}}$ and $\tau_{\mathrm{decay}}^{\mathrm{STH}}$ distributions observed in experimental data (Fig. 6*a*, blue). In contrast, in silico models, which lack such intrinsic variability, produced significantly narrower distributions. Nonetheless, the experimental rise and decay phases demonstrate excellent fit quality (*R*^2^ > 0.99; Fig. 6*b*), supporting the stereotyped nature of NBs despite biological heterogeneity. This further justified our choice of a comparative analysis based on STH shape alone, which we regulated by acting on network topology in our simulations (Fig. 6*c*,*d*).

First, we analyzed homogeneous RND, SW, and SF networks. It is worth noticing that SW connectivity (Fig. 2*a*, center) exhibited a degree distribution similar to that of RND networks (Fig. 2*a*, left), as it consisted of a skewed Gaussian distribution, the hallmark of random networks. Also, their STH profiles were very similar, showing comparable values of $\tau_{\mathrm{rise}}^{\mathrm{STH}}$ (Fig. 6*a*, red and orange). Both topologies exhibited a faster rise phase than networks with SF features and with worse fitting performance (*R*^2^ < 0.9; Table 2). SF networks not only achieved better fitting (*R*^2^ > 0.9; Table 2) but also produced values of both $\tau_{\mathrm{rise}}^{\mathrm{STH}}$ and $\tau_{\mathrm{decay}}^{\mathrm{STH}}$ closer to experimental data (Fig. 6*a*, yellow). Nonetheless, it is worth noticing that none of the homogeneous topologies were able to accurately replicate the experimental rising phase.

**Table 2.** Average goodness of fit values relative to the in silico STH profiles

*R* ^2^	RND	SW	SF	SF_1_–RND	SF_2_–RND	SF_3_–RND
Rise	0.88	0.87	0.96	0.98	0.97	0.95
Decay	0.96	0.97	0.96	0.98	0.99	0.95

Better results were obtained by introducing concurrent topologies that featured two distinct degree distributions for incoming and outgoing connections. Specifically, we used a SF distribution for the indegree (for which we tested different $\alpha_{\mathrm{SF}}$ values) and a RND one for the outdegree. In this type of network, few neurons receive inputs from almost all other neurons, allowing them to regulate the overall network activity. We found that the only configurations that did not show any statistical difference in $\tau_{\mathrm{rise}}^{\mathrm{STH}}$ compared with experimental data were SF–RND with $\alpha_{\mathrm{SF}}=1$ and 2 (SF_1_–RND and SF_2_–RND; Fig. 6*a*). Notably, the SF_2_–RND configuration successfully ensured that both recruitment and relaxation time constants were consistent with in vitro data (Fig. 6*a*). In addition, such time constants revealed an intriguing trend: as $\alpha_{\mathrm{SF}}$ increased, the rise phase shortened, while the decay phase lengthened. In terms of the overall shape of the STH, all SF–RND networks displayed a better fitting than homogeneous connectivity rules, confirming that homogeneous topologies were not the optimal choice to reproduce experimental STH profiles (Table 2). In particular, the SF_2_–RND configuration produced the STH profile that most closely resembled experimental data (Fig. 6*b*) among all considered configurations (Fig. 6*c*,*d*). Additionally, it provided excellent fitting for both the rise and decay phases (*R*^2^ > 0.97 and *R*^2^ > 0.99, respectively; Table 2).

These analyses suggest the temporal profile of population events is mainly shaped by the topological organization of the neuronal network (connectivity), while variations in local parameters mainly determine whether the network operates in a bursting/nonbursting regime. To underpin this finding, we performed an additional systematic analysis using the SF_2_–RND network as a reference (Fig. 6*d*). Starting from the parameter set reported in Table 1, we independently varied 12 critical local parameters related to neuronal membrane and synaptic transmission by ±5 and ±10% around their reference values (Table 3). For each condition, which preserved the SF_2_–RND topology, we computed the goodness of fit (*R*^2^) for both the rise and decay phases of its STH and compared these values with those obtained for the reference SF_2_–RND network. After applying multiple-comparison correction, no statistically significant differences were observed (Table 3). These results indicate that varying either constrained or free parameters does not substantially improve in model performance. Moreover, we computed the slopes of both the rise and decay phases of the STH profiles and compared them with experimental data (Table S1). Similarly, no statistically significant differences were found with respect to experimental values after correction for multiple testing.

**Table 3.** Goodness of fit (*R*^2^) *p* values (with mean deviation, %) of the STH obtained by varying 12 local parameters with respect to the reference SF_2_–RND network, Mann–Whitney *U* test with Benjamini–Hochberg correction

Variation	−10%		−5%		+5%		+10%	
Phase	Rise	Decay	Rise	Decay	Rise	Decay	Rise	Decay
$V_{th}$	0.567 (+0.2%)	0.767 (−0.1%)	0.767 (+0.3%)	1.000 (+0.02%)	0.184 (+1%)	0.400 (−3.9%)	0.184 (+1.1%)	0.200 (−0.9%)
${\bar{g}}_{L}$	0.184 (+0.5%)	1.000 (+0.1%)	0.620 (+0.3%)	0.932 (−0.01%)	0.567 (+0.6%)	0.184 (−21%)	0.767 (+0.8%)	0.184 (−2.2%)
${\bar{g}}_{Na}$	1.000 (−0.1%)	0.200 (−3.1%)	0.200 (+0.9%)	0.767 (−0.2%)	0.184 (+0.5%)	0.400 (−0.4%)	0.400 (+0.5%)	0.400 (−0.7%)
${\bar{g}}_{K}$	0.567 (+0.4%)	0.767 (+0.1%)	0.200 (−1%)	0.932 (+0.1%)	0.400 (+0.7%)	0.324 (−1%)	0.184 (+0.7%)	0.184 (−1.3%)
$\sigma_{N}$	No NBs	No NBs	No NBs	No NBs	0.948 (+0.04%)	0.697 (−0.4%)	0.200 (+0.5%)	0.324 (−0.5%)
${\bar{g}}_{AMPA}$	0.361 (−0.1%)	0.507 (−0.5%)	0.767 (−0.4%)	0.767 (−0.1%)	0.184 (+0.6%)	0.767 (−0.2%)	0.200 (+0.6%)	0.767 (−0.3%)
$\tau_{AMPA}$	0.200 (+0.9%)	0.620 (−0.4%)	0.200 (+1.3%)	0.446 (−0.4%)	0.184 (+0.7%)	0.324 (−0.6%)	0.184 (+0.6%)	0.767 (−0.2%)
${\bar{g}}_{NMDA}$	0.400 (+0.6%)	0.697 (−2.8%)	0.767 (−0.6%)	0.767 (−2.8%)	0.567 (+0.4%)	0.697 (−1.4%)	0.324 (+0.6%)	0.697 (−0.4%)
$\tau_{decay}^{NMDA}$	0.184 (+0.9%)	0.767 (−0.8%)	0.200 (+0.9%)	0.767 (−3%)	1.000 (+0.6%)	0.400 (−3.8%)	1.000 (+0.2%)	0.567 (−0.6%)
$\tau_{rise}^{NMDA}$	0.400 (+0.5%)	0.400 (+0.4%)	0.767 (+0.4%)	0.620 (−1%)	0.567 (+0.2%)	0.400 (−0.7%)	0.200 (+0.4%)	0.324 (−1.7%)
$f_{D}$	No NBs	No NBs	No NBs	No NBs	No NBs	No NBs	No NBs	No NBs
$\tau_{STD}$	No NBs	No NBs	No NBs	No NBs	No NBs	No NBs	No NBs	No NBs

Therefore, these additional analyses confirm our observation that while local parameters regulate whether bursting activity emerges, the temporal structure of network-wide events is primarily determined by network topology, with local parameters contributing only minor quantitative modulations.

### The hierarchical organization of afferent connections governs the interplay between structure and dynamics

In the previous section, we analyzed how the topological characteristics of the network influenced the overall activity and shaped the average NB profile. In this section, we focus on investigating the intricate relationship between structural connectivity and dynamic behavior.

Firstly, we performed a PCA on the IFR traces to identify the most significant data patterns, quantifying the relative contribution of individual neurons to the overall dynamics of the system. In line with their inherently variable nature (see above), in vitro activity exhibited high dimensionality, with the first PC on average accounting for 31 ± 14% of the explained variance with considerable variability. Also, on average more than 6 PCs were needed to explain 50% of the variance (Fig. S6*a*). Differently, in silico activity was notably low dimensional (Fig. S6*b*) and with less variability (a difference already observed for the $\tau_{\mathrm{rise}}^{\mathrm{STH}}$ and $\tau_{\mathrm{decay}}^{\mathrm{STH}}$ distributions). Specifically, the first PC accounted for over 82% of the variance across all configurations, while the second component contributed <3% (Fig. S6*b*). Therefore, we focused our analysis exclusively on the first PC and evaluated the weight of each neuron $\left(w_{n}^{\mathrm{PCA}}\right)$ by means of Eq. 13. We then assessed the relationship between the dynamics of collective activity and the structure of the network by comparing $w_{n}^{\mathrm{PCA}}$ for each neuron n with its connectivity degree, distinguishing between its incoming $\left(\deg_{n}^{\mathrm{IN}}\right)$ and outgoing $\left(\deg_{n}^{\mathrm{OUT}}\right)$ connections.

**Figure 7.**
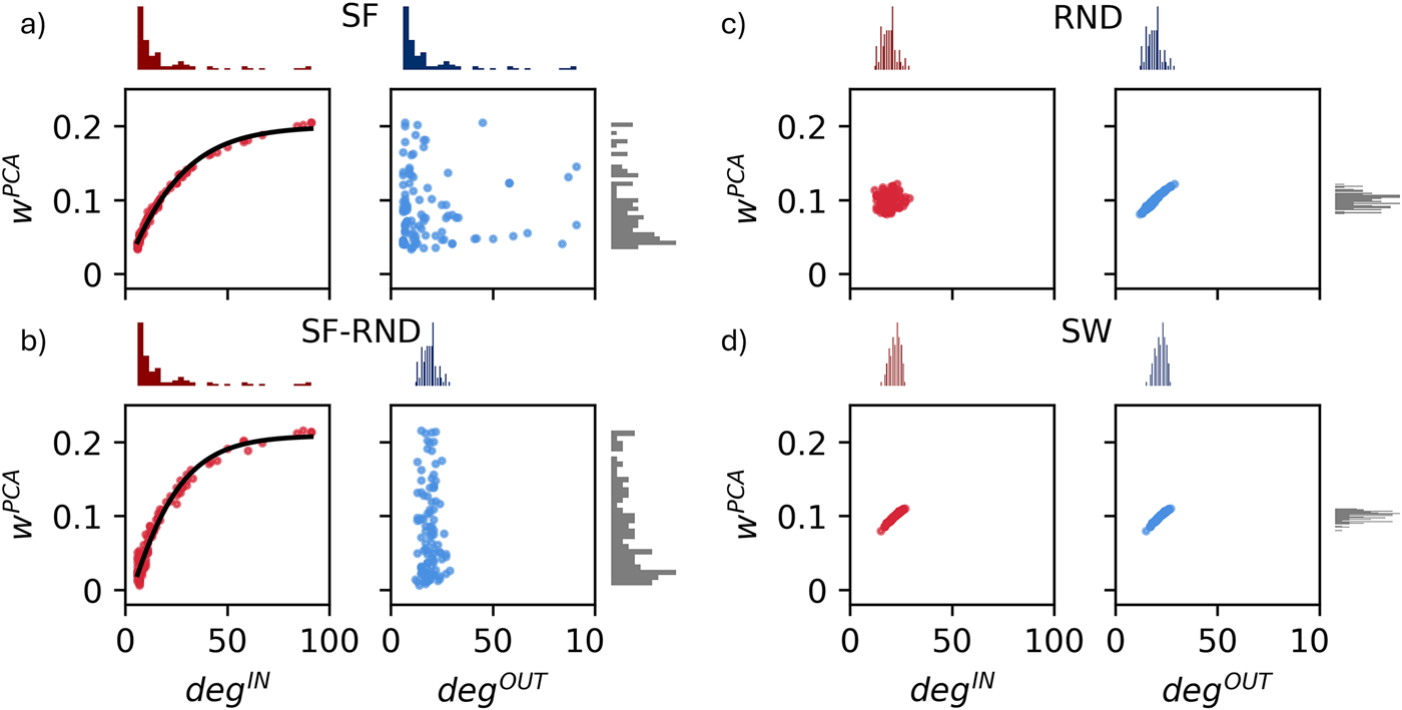
Relationship between structural connectivity and dynamics. The weight of the first PC $\left(w_{n}^{\mathrm{PCA}}\right)$ extracted from the PCA of each neuron's IFR is analyzed in relation to structural connectivity $\left(\deg_{n}^{\mathrm{IN}/\mathrm{OUT}}\right)$ for four network topologies: (***a***) SF, (***b***) SF–RND (*α*_SF_ = 2), (***c***) RND, and (***d***) SW. Incoming (red) and outgoing (blue) connections are distinguished. Marginal histograms of the connectivity degree (top) and first PC weights (right) are displayed alongside each plot. The black curve represents a sigmoid fit (Eq. 13).

In Topological organization influences the temporal profile of population-wide burst dynamics, we showed that SF topologies, whether homogeneous or heterogeneous, produced the most in vitro-like STH profiles. This suggested that SF properties better match the underlying mechanisms driving network activity. We observed that also the $w_{n}^{\mathrm{PCA}}$ distribution was wide and skewed, resembling the SF degree distributions. This held both in the homogeneous (Fig. 7*a*) and in the heterogeneous SF–RND networks (Fig. 7*b*; Fig. S7), where the $w_{n}^{\mathrm{PCA}}$ distribution followed the $\deg_{n}^{\mathrm{IN}}$ (i.e., SF) distribution rather than the $\deg_{n}^{\mathrm{OUT}}$ (i.e., RND). Accordingly, we observed that $w_{n}^{\mathrm{PCA}}$ showed a marked correlation with $\deg_{n}^{\mathrm{IN}}$, but no correlation with $\deg_{n}^{\mathrm{OUT}}$ (Table S2). Additionally, the relationship between $w_{n}^{\mathrm{PCA}}$ and $\deg_{n}^{\mathrm{IN}}$ was not linear; rather, it exhibited a saturating trend (Fig. 7*a*,*b*; Eq. 13). The robustness of this result was highlighted by the consistency of the trend across configurations involving SF with varying $\alpha_{\mathrm{SF}}$ (Fig. S7): $\deg_{n}^{\mathrm{IN}}$ showed a strong correlation with $w_{n}^{\mathrm{PCA}}$, while $\deg_{n}^{\mathrm{OUT}}$connections did not.

As in homogeneous SF, the $w_{n}^{\mathrm{PCA}}$ distribution for both RND and SW topologies had a Gaussian-like shape similar to their degree distribution. The correlation between structure and dynamics was still present in RND and SW (Table S2). However, the more constrained degree distributions of these topologies led to more clustered $w_{n}^{\mathrm{PCA}}$ values (Fig. 7*c*,*d*). Moreover, the incoming degree distribution of RND networks was highly scattered and uncorrelated with $w_{n}^{\mathrm{PCA}}$ (Fig. 7*c*), further emphasizing the inadequacy of this topology for describing in vitro neuronal network connectivity (van den Heuvel and Sporns, 2011; Downes et al., 2012; Poli et al., 2015). In contrast, SW networks demonstrated a stronger correlation between $\deg_{n}^{\mathrm{IN}}$ and $w_{n}$, making them a more suitable choice than RND (Fig. 7*d*). Nevertheless, SF topologies, particularly for afferent connections, remained the most effective option for capturing the desired dynamics. The marked correlation between the incoming degree $\deg_{n}^{\mathrm{IN}}$ and the network’s activity $w_{n}$ can be interpreted in light of theprevious findings, where we showed that the SF connectivity rule for incoming degree $\left(\deg_{n}^{\mathrm{IN}}\right)$ yielded an STH profile (i.e., average NB firing profile) that most closely matched the experimental data (Fig. 6).

## Discussion

In the present work, we developed an in silico model to investigate the interplay between the spontaneous dynamics of excitatory hiPSC-derived neuronal networks cultured over MEAs and the properties of individual neurons, as well as the complexity of network organization.

A defining property of in vitro neuronal cultures, including hiPSC-derived networks, is the alternation of spiking and network bursting activity. Therefore, we first ensured that our model could robustly reproduce this hallmark behavior. In recent computational studies, this distinctive dynamics is often described by macroscopic parameters (e.g., bursting rate), which overlook their full complexity. Here, we used the STH as target feature because it indicates spatiotemporal integration in neuronal activity and offers deeper insight into population recruitment and relaxation. We adopted a conductance-based HH model rather than a simpler phenomenological model (e.g., Izhikevich), not only for its ability to reproduce the cellular biophysical mechanisms and the skewed and heavy-tailed spike train histograms (STH) but also for its consequent suitability for studying pharmacological manipulations (Pospischil et al., 2008). We adapted our framework from a validated human neuronal model (Doorn et al., 2023), ensuring that the core biophysical mechanisms are grounded in experimental observations and specifically tailored to the dataset presented in Parodi et al. (2023). Importantly, we focused exclusively on excitatory networks as, currently, GABAergic hiPSC-derived neurons are less characterized than their glutamatergic counterparts. Future work could extend the model by incorporating inhibitory neurons to study E/I balance once reliable inhibitory neuron data becomes available.

### Balancing local parameter complexity with model robustness in network simulations

We followed a parsimonious design principle, progressively excluding redundant parameters that did not improve quantitative agreement with experiments. For example, additional adaptation currents or short-term facilitation processes were excluded once they failed to improve STH fitting. The remaining free parameters were tuned to reproduce key experimental metrics, capturing essential network features without unnecessary complexity. Their manipulation enabled exploration of how parameter changes modulate network dynamics and transitions between different dynamic states. This approach reduced the effective dimensionality of the parameter space and improved model interpretability and robustness.

Consistent with previous studies (Orlandi et al., 2013; Hernández-Navarro et al., 2021), our findings support the notion that NBs are intrinsically noise-driven phenomena (Supplementary Information). Moreover, our simulations revealed that excitatory neuronal networks display an imbalance in the excitatory drive that sustains activity. While AMPA conductance strongly influences network activation, it imposes a narrow viable boundary (Fig. S4). In contrast, NMDA receptor–mediated currents play a critical role in shaping BD and excitability within a comparatively stable range, making them more scalable and operationally dominant in controlling burst structure (Figs. 4,5). This result has intriguing implications for translational research. To reproduce human-based culture activity, we minimized AMPA conductance within the murine range (Destexhe et al., 1998; Fig. S4), and we tuned NMDA conductance to regulate excitability (Figs. 4,5). Notably, NMDA receptor activity is closely associated with memory consolidation and learning processes (Collingridge, 1987; Castellano et al., 2001; Li and Tsien, 2009). This might suggest a mechanism by which human neurons achieve enhanced spatiotemporal integration compared with murine ones (Cajal et al., 1995; DeFelipe, 2011; Mohan et al., 2015; Deitcher et al., 2017; Zhang et al., 2021), potentially providing an explanation for their greater “computational power” (Masoli et al., 2024).

Short-term synaptic depression served as the complementary modulator for network silencing. We regulated network activity by balancing the magnitude of depression and NMDA conductance, defining an optimal operating point that accurately reproduces in vitro single-channel metrics (Fig. 5). At the same time, the presence of multiple parameter sets capable of generating qualitatively similar bursting dynamics (Fig. 4) reflects a degree of degeneracy intrinsic to nonlinear neuronal systems. Such nonuniqueness highlights that NBs are a robust emergent property of excitation and noise dynamics, rather than the artifact of specific parameter values. While this intrinsic flexibility makes parameter identification more difficult, it also suggests that the model could be adapted to account for variability across different experimental preparations. Although capturing all heterogeneity within a single model remains challenging, personalized parameter tuning could better reflect culture- or patient-specific signatures. Complementary validation efforts will also be needed to test the model beyond the conditions represented in the dataset. Extending the model to pharmacological or electrical manipulations would provide a valuable next step to evaluate its predictive power and refine its parameter space.

### Network topology as a key determinant of emergent network dynamics

While local parameters had minimal influence on the STH, mainly affecting single-channel metrics (see above, Balancing local parameter complexity with model robustness in network simulations), global topology greatly shaped these profiles (Fig. 6; Table 2). Not all simulated architectures sustained in vitro-like NBs: homogeneous RND, SF, and SW topologies failed in the task, whereas concurrent topologies with distinct incoming and outgoing connections reproduced the experimental STH profiles.

A particularly noteworthy trend emerged regarding the slope of the scale-free networks $\left(\alpha_{\mathrm{SF}}\right)$ in concurrent SF–RND topologies (Fig. 6). This parameter governs the number of hubs within the network, thereby influencing recruitment dynamics and burst characteristics. Under constant average connectivity, higher $\alpha_{\mathrm{SF}}$ values produce fewer hubs, but the remaining neurons still receive many afferences (Fig. S8). Lacking a dampening circuit with few connections, this topology accelerates network recruitment and prolongs decay. Conversely, lower $\alpha_{\mathrm{SF}}$ values increase the number of both hubs and poorly connected neurons (Fig. S8), resulting in shorter, less widespread bursts. The topology that best matched the experimental STH profile featured a SF incoming connectivity with intermediate slope $\left(\alpha_{\mathrm{SF}}=2\right)$ and a RND outgoing organization (Fig. 6; Table 2; Fig. S7). This indicates that a balanced distribution of hubs and low-degree neurons is crucial for accurate spatiotemporal integration. In this configuration, the SF organization of afferent connections implies that a subset of “privileged” neurons, the hubs, receives the majority of inputs, effectively acting as central activity regulators. NBs are generally believed to arise when excitability crosses a critical threshold (Orlandi et al., 2013; Kumar et al., 2020) driven by a gradual buildup through excitatory recurrent feedback. Consistently, our simulations show that hubs behave as pacemakers with sustained tonic firing. However, unlike intrinsically active pacemakers (Gritsun et al., 2010), our hubs emerge from the structural organization of the networks, highlighting that network events are emergent structural properties rather than intrinsic neuronal characteristics.

To further explore the relationship between topological properties and network dynamics, we analyzed the correlation between the imposed structural connectivity and network’s activity, evaluated with PCA. Homogeneous RND networks showed no correlation between indegree and network activity (Fig. 7; Table S2), emphasizing their limited ability to capture the system dynamics and indicating that this topology may be less effective in reproducing collective network activity. In vitro 2D networks develop mesoscale architectures that deviate from pure randomness, and such self-organization may influence both the initiation and richness of spontaneous dynamics (Okujeni et al., 2017; Antonello et al., 2022). Although SW networks support robust and efficient communication between distinct areas (Sporns and Betzel, 2016), their modularity contrasts with the integrative nature of in vitro networks (Poli et al., 2016). Therefore, SW topologies may still be inadequate for in vitro modeling, where structural homogeneity hampers functional specialization. In our simulations, SW networks outperformed RND, exhibiting correlations between dynamic weights and both degrees, but only within a narrow range (Fig. 7; Table S2). This suggests a less generalizable and robust relationship that makes SW a suboptimal topology. Functional connectivity analyses of in vitro neuronal networks have frequently highlighted a SF and hierarchical organization (Eytan and Marom, 2006; Brofiga et al., 2022). Within these networks, hubs are believed to play a pivotal role in efficient information transfer (van den Heuvel and Sporns, 2013). Accordingly, we found strong correlations between indegree and network activity in all SF networks, negligible for outdegree (Fig. 7; Fig. S7; Table S2). This highlights the dominant role of incoming connectivity: neurons integrating many inputs exert greater influence on population activity than those with many outputs. The broad degree distribution in SF networks underscores the robustness of this result, positioning SF networks as the most plausible topology. Additionally, the saturating trend at high indegree values (Fig. 7; Fig. S7) suggests a form of homeostatic regulation preventing unbounded activity escalation with increased connectivity degree.

Because the detailed structural organization of in vitro cultures is largely inaccessible, the topologies investigated in this study should be regarded as idealized abstractions. In this context, our approach represents a modeling strategy to link experimentally observed mesoscale features with simplified network models. Rather than suggesting that in vitro networks strictly conform to a specific topology, our results indicate that the concurrent organization may approximate key structural and functional principles observed experimentally.

Overall, our findings highlight the crucial role of topology—particularly incoming connectivity—in shaping emergent dynamics. SF structures naturally generate hubs that coordinate global activity and burst initiation, emphasizing that structural properties, rather than local neuronal dynamics, drive collective network behavior.
